# Complex Virus–Host Interactions Involved in the Regulation of Classical Swine Fever Virus Replication: A Minireview

**DOI:** 10.3390/v9070171

**Published:** 2017-07-05

**Authors:** Su Li, Jinghan Wang, Qian Yang, Muhammad Naveed Anwar, Shaoxiong Yu, Hua-Ji Qiu

**Affiliations:** State Key Laboratory of Veterinary Biotechnology, Harbin Veterinary Research Institute, Chinese Academy of Agricultural Sciences, 678 Haping Road, Harbin 150069, China; lisu@hvri.ac.cn (S.L.); wangjinghan1278@163.com (J.W.); yangqian@hvri.ac.cn (Q.Y.); dr.naveed903@gmail.com (M.N.A.); yushaoxiong1987@hotmail.com (S.Y.)

**Keywords:** classical swine fever virus, virus–cell interactions, attachment, entry, cell apoptosis, virus life cycle

## Abstract

Classical swine fever (CSF), caused by classical swine fever virus (CSFV), is one of the most devastating epizootic diseases of pigs in many countries. Viruses are small intracellular parasites and thus rely on the cellular factors for replication. Fundamental aspects of CSFV–host interactions have been well described, such as factors contributing to viral attachment, modulation of genomic replication and translation, antagonism of innate immunity, and inhibition of cell apoptosis. However, those host factors that participate in the viral entry, assembly, and release largely remain to be elucidated. In this review, we summarize recent progress in the virus–host interactions involved in the life cycle of CSFV and analyze the potential mechanisms of viral entry, assembly, and release. We conclude with future perspectives and highlight areas that require further understanding.

## 1. Introduction

Classical swine fever (CSF), which is caused by classical swine fever virus (CSFV), is a severe and highly contagious disease in pigs that is listed by the World Organization for Animal Health (OIE). The disease is distributed in many countries and areas including Asia, Eastern Europe, Russia, and South America [1]. Currently, CSF is prevented by stamping-out (non-vaccination) and systemic prophylactic (vaccination) policies [2]. In China, vaccines based on C-strain, a lapinized live attenuated vaccine strain, have been widely used to control CSFV infections in the pig population. Therefore, large-scale outbreaks have been rarely observed in the field during the past decades. However, annual sporadic epizootics or endemics in some regions are continuously being observed. A mild, atypical form of the disease with a long duration, atypical clinical signs, and relatively low morbidity and mortality has been observed constantly, even in a proportion of vaccinated pigs [3]. Based on the phylogenetic analysis of nucleotide sequences, there are three genotypes of CSFV isolates, which can be further divided into 11 subgenotypes. So far, there is no clear correlation between specific sequence motifs and the virulence of the different field strains [4,5].

**Figure 1:**
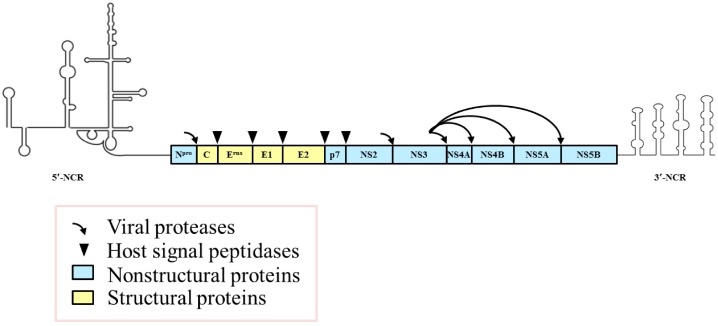
The organization of the classical swine fever virus (CSFV) genome and the encoding proteins. The positive-sense, single-stranded RNA genome of 12.3 kb contains 5′- and 3′-noncoding regions (NCRs) important for viral RNA replication and/or protein translation and a large open reading frame (ORF) that encodes a large polyprotein. The polyprotein is processed into four structural proteins and eight nonstructural proteins by a combination of viral and cellular proteases.

CSFV is an enveloped, positive-sense, single-stranded RNA virus, which belongs to the *Pestivirus* genus of the *Flaviviridae* family [6]. The genome of CSFV contains a 5′-noncoding region (5′-NCR), a large open reading frame (ORF), and a 3′-NCR. The ORF is translated into a precursor polyprotein of 3898 amino acids (aa), which is cleaved into four structural proteins (C, E^rns^, E1, and E2) and eight non-structural proteins (N^pro^, p7, NS2, NS3, NS4A, NS4B, NS5A, and NS5B) (Figure 1) [7]. CSFV enters the host through the mucous membranes of the oral and nasal cavities, and initially infects cells of the tonsil, then spreads around the body via the blood and lymph circulation. CSFV has a distinct tropism for cells of the immune system, which causes severe leukopenia that is associated with apoptosis of leukocytes in the thymus, spleen, lymph nodes, and bone marrow of infected pigs [8,9]. The eventual outcomes of virus infection are generally associated with complex and multifaceted host responses to the virus.

This review aims to summarize recent progress in the virus biology and virus–host interactions at the interface of virus replication, and highlight potential mechanisms in the CSFV life cycle. The review concludes with future perspectives and highlights areas that require further understanding.

## 2. The CSFV Envelope Proteins Mediate Virus Attachment and Entry

The structural components of the CSFV virions include a capsid protein (C) and three envelope glycoproteins (E^rns^, E1, and E2). The glycoproteins are processed from the precursor E^rns^-E1-E2 by the host signal peptidase. The E^rns^ protein consists of 227 aa with a molecular weight of around 48 kDa, which is glycosylated with carbohydrate moieties at seven glycosylation sites [10]. Due to the unusual C terminus, the protein is loosely associated with mature virions and is also secreted into the medium of cultured infected cells. In general, E^rns^ is present as a homodimer (with a molecular mass of about 100 kDa) [11] and a heterodimer with E2 [12] on the virion. The ectodomain of E^rns^ contains five α helices and seven β strands with a concave and a convex face and is stabilized by four intramolecular disulfide bonds [13]. In addition, structural analyses of the C-terminus of E^rns^ show that the amphipathic α-helix is inserted slightly tilted into the membrane [14]. E^rns^ possesses ribonuclease activity, induces lymphocytes apoptosis, and antagonizes the response of type I interferon (IFN) signaling. In addition, the interaction between E^rns^ and membrane-associated heparan sulfate (HS) [15] or laminin receptor (LamR) [16] mediates virus attachment. CSFV cultured in swine kidney cells (SK6 cells) selects a virus variant (with S476R mutation) of E^rns^ that attaches to the surface of cells by interacting with HS [15]. 

The E1 glycoprotein consists of 195 aa with an apparent molecular mass of 33 kDa, which contains three *N*-linked putative glycosylation sites and six cysteine residues. E1 is a type I transmembrane protein with an N-terminal ectodomain and a C-terminal hydrophobic anchor that attaches E1 to the envelope of the virus [11]. E1 and E2 form heterodimers via disulfide bridges between cysteine residues that are present in the CSFV virions. The formed heterodimers then mediate the process of viral entry [17,18].

**Figure 2:**
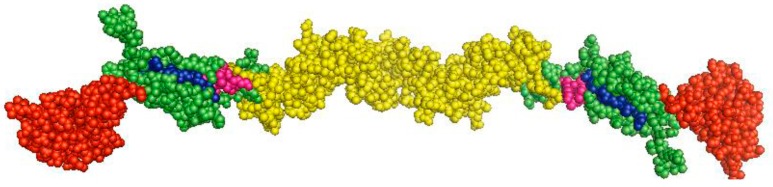
Predicted three-dimensional structure of the CSFV E2 protein. Homology modeling analysis of the CSFV E2 protein was performed using the software PyMOL 1.7 according to the structure of the BVDV E2 protein. Domains B/C are shown in red, domains D/A in green, the other region in yellow, and the fusion peptides (FP1 and FP2) of E2 in blue or purple.

The E2 protein is a 55-kDa glycoprotein that consists of 373 aa, and contains six N-linked and one O-linked putative glycosylation sites. E2 possesses an N-terminal signal peptide and a C-terminal transmembrane domain that anchors E2 to the viral envelope. The CSFV E2 protein forms disulfide-linked homodimers with molecular weights of 100 kDa. E2 is the most immunogenic of the CSFV glycoproteins, in terms of inducing neutralizing antibodies and protection against lethal virus challenge [19,20,21,22]. Removal of the glycosylation sites of E2 can significantly reduce the immunogenicity of the protein [23]. Antigenic mapping of E2 has been determined that attributes to domains A to D using a panel of monoclonal antibodies (MAbs) [24]. The antigenic epitopes of domains D/A, but not the domains B/C, are the most conserved epitopes. A highly conserved neutralizing linear epitope in the domain A, 829TAVSPTTLR837, which is recognized by the MAb WH303, has been identified [25]. The epitope is widely used to develop marker vaccines [26,27,28] and diagnostic assays [27,29]. However, the crystal structure of the CSFV E2 protein has not been resolved so far, which renders it difficult to map conformational epitopes on the protein. The CSFV E2 protein shares a sequence identity of 65% with the bovine virus diarrhea virus (BVDV) E2 protein. Recently, the crystal structure of the BVDV E2 has been resolved, which can be divided into three domains (I to III) [30]. Comparative analysis of the E2 proteins revealed that domains I and II of BVDV correspond to CSFV antigenic domains B/C and D/A, respectively. E2 is characterized as a class II fusion protein that harbors two fusion peptides, ^818^CPIGWTGVIEC^828^ and ^869^CKWGGNWTCV^878^ (Figure 2). Interestingly, the peptides exert membrane fusion activity and play critical roles in viral replication and virulence [31,32]. The mechanism of the fusion process of pestiviruses has not been fully elucidated. Based on the crystal structure of the BVDV E2 protein, Li and his colleagues presumed three potential fusion mechanisms for pestiviruses: (a) the aromatic residues in domain IIIc of E2 function as a fusion motif, (b) domain I of E2 contains a fusion motif, and (c) E1 contains the fusion motif and E2 functions as a coeffector [30]. Another study has also resolved the structure of the BVDV E2 protein and presumed that E2 becomes disordered at low pH and exposes the fusion loop of E1, thus mediating the fusion between viral envelope and endosome membrane [18]. In addition, several host cellular factors have been shown to be associated with E2 and are involved in the CSFV life cycle, e.g., CD46 has been identified as a receptor for BVDV using an anti-E2 idiotypic antibody [33], which also functions as an important factor for the attachment of CSFV [34]. Host factors that mediate viral attachment have been defined, but the functional receptor(s) of CSFV has not been determined, and the process of fusion should be focused on future studies.

## 3. Modulation of Viral Genomic Replication and Translation by NCRs and Nonstructural Proteins (NSPs)

The 5′- and 3′-NCRs of CSFV, approximately 373 and 228 nucleotides (nt) in length, respectively, form stem-loops at the N- and C-termini of the genome [7]. The 5′-NCR does not contain the cap structure, but harbors an internal ribosome entry site (IRES) to initiate cap-independent translation. The 3′-NCR, lacks a poly(A) tail but contains a variable AU-rich region and a conserved region, is involved in the initiation of viral genome replication [35]. The NSPs of CSFV consist of N^pro^, NS2, NS3, NS4A, NS4B, NS5A, and NS5B. N^pro^, NS2, NS3, and NS4A have been shown to be involved in the cleavage of the NSPs. A previous study has shown that NS3, NS4A, NS4B, NS5A, and NS5B are required for CSFV replication [36]. NS2, a transmembrane protein, harbors an auto-protease activity that is responsible for *cis*-cleavage of NS2-3 [37]. Previous studies have shown that the uncleaved NS2-3 is crucial for the generation of infectious viral particles for CSFV or BVDV [38,39]. However, additional evidence suggests that the uncleaved NS2-3 is not required for the virion morphogenesis of pestiviruses [40]. As a multifunctional protein, NS3 acts as serine protease, helicase, and nucleoside triphosphatase (NTPase) [41,42,43]. NS3 and its cofactor, NS4A, process all downstream cleavage sites of viral NSPs [44]. The structure of the NS3-NS4A complex reveals surface interactions between the NS3 protease domain and NS4A-kink region that is required for RNA replication and replicase assembly [45]. NS4B contains two conserved domains, Walkers A (aa 209–216) and B (aa 335–342). Walker A exhibits NTPase activity and is essential for RNA replication [46]. Analysis of simple modular architecture research tool (SMART) has revealed that NS4B contains a Toll/interleukin-1 receptor (TIR)-like domain, and mutations in the TIR-like domain of NS4B significantly attenuate the virulence of CSFV in pigs [47]. The CSFV NS5A contains the conserved sequence C^2717^-C^2740^-C^2742^-C^2767^, which forms the zinc-binding motif that is required for viral RNA synthesis and viral growth. The NS5A protein of BVDV or hepatitis C virus (HCV) is a highly-phosphorylated protein [48]. Similarly, several potential phosphorylated sites of the CSFV NS5A can also be found using the bioinformatic analysis (NetPhos 3.1 Server). It has been reported that NS5A can induce the autophagy pathway of host cells and enhance viral replication [49]. A recent study shows that the inhibition of autophagy promotes apoptosis in CSFV-infected cells via the reactive oxygen species (ROS)-dependent retinoic acid inducible gene I (RIG-I)-like receptor signaling pathway [50]. NS5B is an RNA-dependent RNA polymerase (RdRp) that harbors a conserved motif GDD, which is in charge of RNA replication [51]. The structure of pestiviral NS5B proteins resembles a right hand with fingers, palm, and thumb domains, thus exhibiting the typical general fold of RdRp [52]. It has been shown that NS3, NS4A, NS4B, NS5A, and NS5B are sufficient for the genome replication [36]. The interactions between NSPs and NCRs have been determined to be involved in modulation of RNA replication and translation [53,54,55].

The CSFV genome can be transcribed into negative-strand RNA that can be used as the template to produce the positive-strand RNA. During this process, NS5B binds to the negative-strand RNA to produce more positive-strand RNA copies [44]. Moreover, NS3 interacts with NS5B and enhances the NS5B RdRp activity through its N-terminal protease domain. NS5A regulates viral RNA synthesis through interacting with NS5B and 3′-NCR [56]. When NS5A is present at a lower expression level in the cells, it preferably interacts with NS5B and enhances viral RNA replication. But oversaturated NS5A will interact with 3′-NCR and thus inhibit viral RNA replication [56]. It is likely that CSFV modulates RNA replication via the regulation of NS5A expression.

Unlike cellular mRNA, the CSFV genome lacks 5′-terminal cap structure, and the IRES located in the 5′-NCR can be recognized by the ribosome to initiate translation [57]. NS3 can bind to IRES and promote IRES-mediated translation [54]. In comparison with NS3, NS5A inhibits the IRES-mediated translation, whereas NS5B can suppress the effect of NS5A on the IRES [55]. In addition, NS5B can stimulate NS3 to increase the efficiency of viral genome translation [54].

## 4. Interactions between CSFV and Host Cellular Proteins Are Necessary for the CSFV Life Cycle

**Figure 3:**
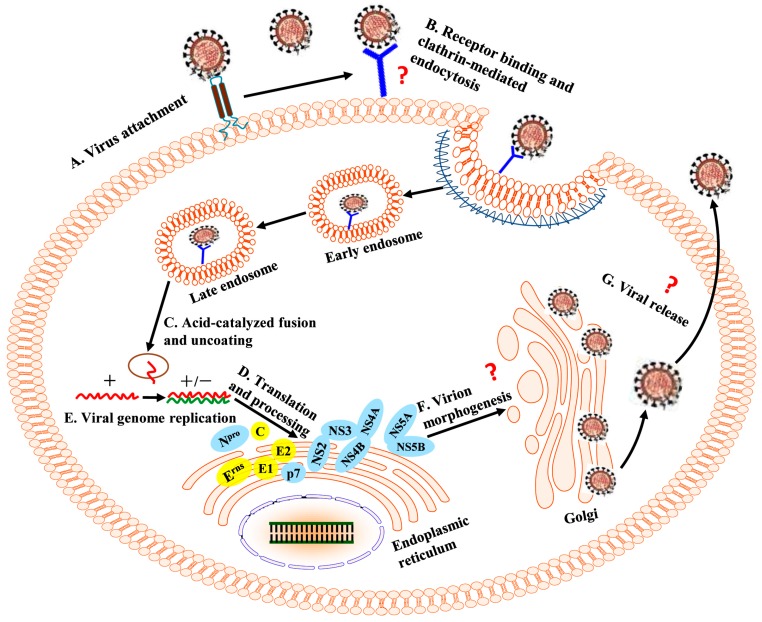
Schematic diagram of the CSFV life cycle. (**A**) Interactions between E^rns^ and host cellular heparan sulfate (HS) and/or laminin receptor (LamR) mediate virus attachment. (**B**) Virus binds to an unknown entry receptor(s) and triggers clathrin-mediated endocytosis. (**C**) Low pH facilitates viral envelope and membrane fusion. (**D**) Translation and processing of viral proteins. (**E**) Viral genome replication. (**F**) Virion morphogenesis harbors an unknown strategy. (**G**) Mature virions are released from the cell via an unknown secretory pathway. +, positive-strand genomic RNA; +/−, positive- and negative-strand replicative intermediate.

During CSFV infection, interactions between the virus and HS/LamR mediate virus attachment [15,16]. Subsequently, virus entry is a dynamin-, and cholesterol-dependent, and clathrin-mediated endocytosis that requires Ras-related in brain (Rab) 5 and Rab7 [58]. The fusion between cellular membrane and viral envelope is pH-dependent and is triggered by the acidification of the endosome. Another pestivirus, BVDV entry also requires clathrin-mediated endocytosis and low endosomal pH [59]. Similarly, Rab5 and Rab7 are involved in the life cycles of HCV [60], dengue virus (DENV) [61], and West Nile virus [62] that belong to the family *Flaviviridae*. It has been demonstrated that the peptides ^129^CPIGWTGVIEC^139^ and ^180^CKWGGNWTCV^189^ of the CSFV E2 protein mediate fusion between viral envelope and cellular membrane [31,32] (Figure 2). After uncoating, the viral genome is released and translated into the viral proteins, followed by the cleavage of the cellular and viral proteases. In addition, the viral genome can be transcribed into negative-strand RNA, which is used as a template to produce progeny positive-sense RNA. Virion morphogenesis is mediated by NS2-3 and NS4A [38]. Then, the virion is released from the host cells (Figure 3). Host cellular factors also participate in various steps of the life cycle of CSFV.

### 4.1. Host Factors Modulate the Production of Progeny Virus

The interactions between flaviviruses and cytoskeleton are involved in the entry, transport, assembly, and egress processes [63]. The cellular β-actin interacts with the E2 protein and affects the early stage of the replication cycle of CSFV [64], which is most likely related to the interaction affects intracellular transport process of CSFV or E2 protein in the cell at the post-entry step. Annexin A2 (Anx2) is a lipid raft-associated scaffold protein that functions in membrane trafficking, aggregation of vesicles, and endosome formation. Anx2 is involved in the regulation of the life cycles of many viruses, such as cytomegalovirus [65], human immunodeficiency virus type 1 [66], influenza virus [67], and HCV [68]. Anx2 interacts with E2 and promotes CSFV production [69], and treatment of PK-15 cells with Anx2-specific polyclonal antibody significantly inhibited CSFV growth, thus we presume that Anx2 likely participates in the virus attachment or entry. In addition, interaction between Anx2 and NS5A enhances the virus assembly rather than in genome replication and virion release [70]. It is possible that Anx2 participates in the multiple steps of the CSFV life cycles. The interaction between C and osteosarcoma amplified protein 9 (OS9) inhibits the virus replication in the cell culture [71]. Host factors also affect NS5A-regualted viral genome synthesis and translation, e.g., heat shock protein 70 (HSP70) interacts with NS5A and promotes viral RNA replication [72]. Furthermore, eukaryotic elongation factor 1A (eEF1A) has been shown to interact with NS5A of CSFV and inhibit IRES-mediated translation efficiency [73] (Table 1). eEF1A also binds to the NS5A protein of BVDV. However, the effect of eEF1A on the BVDV replication remains unclear [74]. It is plausible to speculate that eEF1A is a broad host factor that interacts with the pestiviral NS5A protein.

### 4.2. Viral Proteins Block the Host Innate Immunity

**Figure 4:**
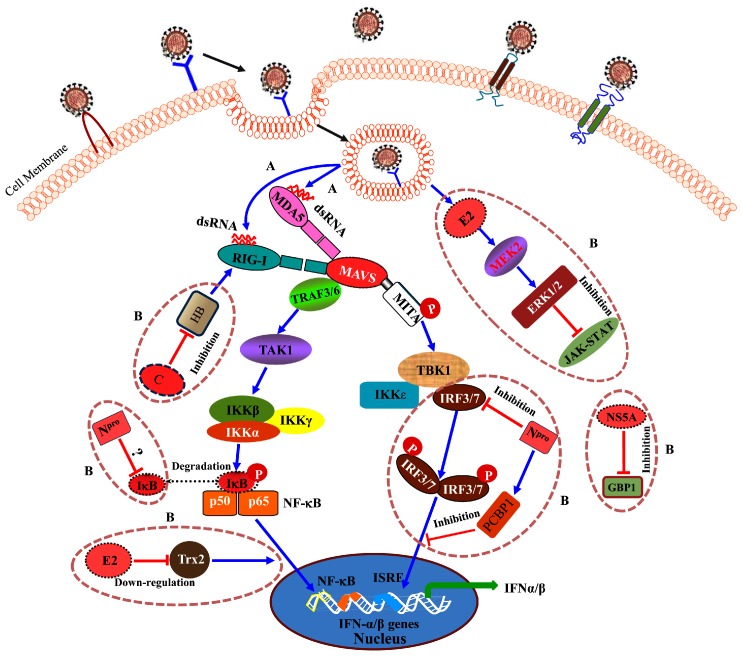
Activation and blockage of the intracellular signaling pathways of innate immunity during CSFV infection. (**A**) CSFV RNA is sensed by melanoma differentiation-associated protein 5 (MDA-5) and retinoic acid inducible gene I (RIG-I). (**B**) CSFV blocks the host innate immunity through multiple steps.

Viruses have evolutionary evolved strategies to evade host innate immune responses for successful virus replication. To facilitate virus infection, CSFV N^pro^ interacts with IFN regulatory factor-3 (IRF-3) or IRF-7 and blocks type I IFN induction [76,77]. The structure of BVDV N^pro^ has been resolved, and the interaction domain harbors a TRASH motif to recognize the immune factors [88]. The host poly(C)-binding protein 1 (PCBP1) negatively regulates the type I IFN pathway and enhances CSFV growth [78]. Hemoglobin subunit beta (HB) interacts with the C protein and antagonizes CSFV replication via the RIG-I-mediated IFN signaling, whereas CSFV inhibits expression of HB to block the pathway [81]. Our recent study has shown that thioredoxin 2 (Trx2) interacts with E2 and negatively regulates CSFV replication via nuclear factor kappa-light-chain-enhancer of activated B cells (NF-κB) signaling, whereas CSFV inhibits protein expression of Trx2 to antagonize the antiviral effects [84]. Another study shows that mitogen-activated protein kinase kinase 2 (MEK2) interacts with the E2 protein and promotes CSFV replication via attenuation of the Janus kinase/signal transducers and activators of transcription (JAK-STAT) signaling pathway [85]. Recently, host guanylate-binding protein 1 (GBP1) has been shown to inhibit CSFV replication depending on its GTPase activity. As an antagonism, CSFV blocks the antiviral activities of GBP1 via inhibition of GBP1 expression [86]. Furthermore, the interaction between N^pro^ and IκBα (the inhibitor of NF-κB) may be involved in the modulation of the NF-κB signaling pathway [79] (Figure 4).

### 4.3. Disruption of Some Virus-Host Interactions Affects the Viral Virulence in Pigs

SUMOylation is a post-translational modification involved in various cellular processes, such as transport, transcriptional regulation, protein stability, cell apoptosis, stress response, and progression of the cell cycle. Viruses have evolved various strategies to evade the host immune response through interacting the cellular SUMOylation pathway [89,90,91], thus destruction of the interaction between virus and host usually attenuates viral virulence [92]. It has been demonstrated that the C protein of CSFV interacts with SUMO-1 (small ubiquitin-like modifier 1) and UBC9 (SUMO-1-conjugating enzyme 9) of the SUMOylation pathway [82]. Intriguingly, the virulence of mutant viruses, which are defective in binding to components of the SUMOylation pathway, is completely attenuated in pigs [82]. The cytoskeleton is required for the life cycle of flaviviruses [63]. As a major cytoskeleton regulator, Ras GTPase-activating-like protein 1 (IQGAP1) interacts with the C protein, and a disruption of such interaction also results in the attenuation of viral virulence [83] (Table 1).

## 5. Changes of Cell Apoptosis and Cell Cycle Induced by CSFV Infection

Acute CSF is associated with high fever, leukopenia, thrombocytopenia, and hemorrhages observed in various organs. During the processes of acute CSF, the virus induces aberrant levels of type I IFN and pro-inflammatory mediators causing a so-called cytokine storm [93,94]. It has been shown that lymphocyte depletion is associated with the strong IFN-α response [94]. In addition, interleukin (IL)-1α, IL-6, and tumor necrosis factor (TNF)-α appeared to be the major cytokines involved in lymphocytopenia [95]. Another study has shown that CSFV infection induces the expression of apoptotic genes, such as CD49d, major histocompatibility complex (MHC) class II, and Fas [8]. Virus components can induce or inhibit apoptosis. Previous studies indicated that E^rns^, 5′- or 3′-NCR of CSFV can induce lymphocyte apoptosis in vivo [96,97]. However, some of the viral proteins, such as N^pro^ and NS2, can inhibit cell apoptosis in vitro [98,99]. As a multi-functional protein, N^pro^ can antagonize the double-stranded RNA-mediated apoptosis [98], whereas, it cannot suppress the apoptosis induced by the NCRs of CSFV [97]. In addition, N^pro^ binds to HS-1-associated protein X 1 (HAX-1, an anti-apoptotic protein) and leads to a redistribution of HAX-1 from the mitochondria to the endoplasmic reticulum (ER), which might increase cellular resistance to apoptosis [80]. The NS2 protein can inhibit MG132-induced apoptosis, and the expression of NS2 results in the cell cycle arrest at S-phase and the induction of ER stress in the swine umbilical vein endothelial cells [99,100]. It is possible that the apoptosis induced by CSFV infection in vivo is associated with the magnitude of cytokine production.

## 6. Concluding Remarks and Prospects

The eventual outcome of viral infection usually relies on the host response to the virus. The virus life cycle consists of attachment, entry, uncoating, biosynthesis, assembly, and release. Attachment factors serve to bind the virion and thus help to concentrate viruses on the cell surface. These factors include HS and other carbohydrate structures on the cell surface. However, the factors usually cannot activate the downstream signals of the host to mediate virus entry. The entry receptor(s) can trigger conformational changes of the virion, activate host signaling pathways, and promote endocytic internalization. The attachment of CSFV is mediated by the host cellular HS or/and LamR [15,16]. As HS or LamR cannot mediate virus internalization, the virus maybe bind to an unknown entry receptor and trigger signaling pathways, such as clathrin-mediated endocytic pathway. CSFV can be internalized by clathrin-mediated endocytosis [58]. Entry of BVDV into Madin–Darby bovine kidney (MDBK) cells also requires active clathrin-dependent endocytosis [59]. However, viruses have evolved divergent strategies to invade host cells, e.g., the entry of influenza virus into simian kidney epithelial cells shows that almost 60% of the particles enter via clathrin-coated pits, whereas 40% use a clathrin-independent pathway [101]. Chlorpromazine, an inhibitor of clathrin lattice polymerization, cannot abrogate the CSFV infection [58], thus we presume that CSFV can be internalized via the clathrin-independent pathway. Furthermore, the low pH facilitates virus membrane fusion [17,58], indicating that the fusion step occurs in the endosome but not the cellular membrane. However, the entry receptors have not been defined, and the detailed entry and fusion mechanisms of CSFV remain to be revealed. Host factors also participate in viral genome replication and translation. Cytoplasmic RNA helicase A (RHA) participates in the modulation of RNA synthesis, replication, and translation of CSFV through binding 5′- and 3′-NCRs [75]. Anx2 has been shown to interact with E2 and NS5A, enhancing viral growth and assembly [69,70]. Thus, we speculate that Anx2 plays critical roles in the multiple phases of the virus life cycle. Furthermore, host heme oxygenase 1 (HO-1) positively regulates CSFV replication [87]. In addition, eEF1A has been demonstrated to modulate viral genome translation through binding to the viral IRES [73]. During virus infection, the cytoskeletal proteins play an essential role in the viral transport and egress processes. The interaction between β-actin and E2 proteins affects the early stage of the replication [64], indicating that β-actin may participate in the transport process of the virus at the post-entry step of the virus life cycle. The process of virus assembly usually involves protein–protein interactions between viral structural proteins and NSPs and the coordinated action of host factors. HCV, DENV, and Japanese encephalitis virus are assembled at ER-derived membranes and exit the cell through the secretory pathway. Host factors, such as Anx2, endosomal sorting complexes required for sorting (ESCRT) components, and Rab18 promote virus assembly, ER budding, and maturation [102]. Anx2 has been shown to interact with NS5A to enhance CSFV assembly [70]. However, detailed dissection of CSFV assembly and release remains to be demonstrated. Taken together, future studies should be focused on the mechanisms of the virus entry, assembly, and release.

Virus infection can trigger a series of signaling cascades in host cells. To establish and maintain persistent infection, the CSFV N^pro^ targets IRF3 and IRF7 to block type I IFN production [76,77], NS5A antagonizes the antiviral activity of GBP1 [86], and C inhibits the RIG-I-mediated IFN-β signaling pathway through interacting with HB [81]. Thus, it seems that CSFV antagonizes the host innate immunity through multiple mechanisms. Novel insights into the mutual antagonism of the virus and host innate immunity will be beneficial for providing valuable targets for virus attenuation. It has been demonstrated that CSFV replicates poorly in cells from MxA-transgenic pigs [103]. More recently, it was reported that the monocytes and macrophages from the genome-edited pigs lacking the scavenger receptor cysteine-rich domain 5 (SRCR5) of CD163 are completely resistant to porcine reproductive and respiratory syndrome virus infection [104]. Dissection of the interplay between CSFV and the host will undoubtedly enrich the understanding of CSFV pathogenesis and facilitate the development of novel strategies for the control and eradication of CSF, such as development of novel antiviral agents, construction of quickly attenuated, efficacious, and highly productive vaccine strains, and generation of CSF-resistant transgenic pigs.

## Figures and Tables

**Table 1:** Interactions between classical swine fever virus (CSFV) and host cellular proteins and replication cycle-contributing factors.

Viral Proteins	Interacting Partners or Replication Cycle-Contributing Factors	Functions	Ref.
5′- and 3′-NCRs	RHA	Modulation of RNA synthesis, replication and translation of CSFV	[75]
N^pro^	IRF-3	Blockage of IFN-β production	[76]
	IRF-7	Blockage of IFN-α production	[77]
	PCBP1	Blockage of IFN-β production	[78]
	IκBα	—	[79]
	HAX-1	Cellular resistance to apoptosis	[80]
C	OS9	Regulation of virus replication	[71]
	HB	Blockage of IFN-β production	[81]
	UBC9	Involvement of viral virulence	[82]
	SUMO-1	Involvement of viral virulence	[82]
	IQGAP1	Involvement of viral virulence	[83]
E^rns^	HS	Attachment receptor	[15]
	LamR	Attachment receptor	[16]
E2	β-Actin	Regulation of virus replication	[64]
	Anx2	Regulation of virus growth	[69]
	Trx2	Inhibition of the NF-κB signaling	[84]
	MEK2	Inhibition of the JAK-STAT signaling	[85]
NS5A	Anx2	Regulation of viral assembly	[70]
	HSP70	Regulation of virus replication	[72]
	eEF1A	Inhibition of IRES-mediated translation efficiency	[73]
	GBP1	Regulation of virus replication	[86]
–	CD46	Involvement of virus attachment	[34]
–	Clathrin	Involvement of virus internalization	[58]
–	Cholesterol	Involvement of virus internalization	[58]
–	Dynamin	Involvement of virus internalization	[58]
–	Rab5	Involvement of virus internalization	[58]
–	Rab7	Involvement of virus internalization	[58]
–	HO-1	Regulation of virus replication	[87]

NCR: noncoding region; RHA: RNA helicase A; IRF: interferon regulatory factor; IκBα: inhibitor of kappa B; HAX-1: HS-1-associated protein X 1; PCBP1: Poly(C)-binding protein 1; IFN: interferon; SUMO-1: small ubiquitin-like modifier 1; UBC9: SUMO-1-conjugating enzyme 9; IQGAP1: Ras GTPase-activating-like protein 1; HB: hemoglobin subunit beta; OS9: osteosarcoma amplified protein 9; HS: heparan sulfate; LamR: laminin receptor; Trx2: thioredoxin 2; NF-κB: nuclear factor kappa-light-chain-enhancer of activated B cells; JAK-STAT: Janus kinase/signal transducers and activators of transcription; Anx2: annexin A2; MEK2: mitogen-activated protein kinase kinase 2; eEF1A: eukaryotic elongation factor 1-alpha 1; HSP70: heat shock protein 70; GBP1: guanylate-binding protein 1; CD46: cluster of differentiation 46; Rab: Ras-related in brain; HO-1: heme oxygenase 1.
